# Supplementary material for: Pervasive RNA-binding protein enrichment on TAD boundaries regulates TAD organization
Source: Nucleic Acids Res. 2025 Jan 7;53(1):gkae1271. doi: 10.1093/nar/gkae1271 (PMC11705077; doi:10.1093/nar/gkae1271)
Supplement: gkae1271_Supplemental_Files [file gkae1271_supplemental_files.zip › NAR_R1_SuppInfo_V1.pdf]

# Inventory of Supplementary Information

## 1. Supplementary Figures and Legends

### Sun Q, Zhou Q et. al. Suppl. Fig. S1

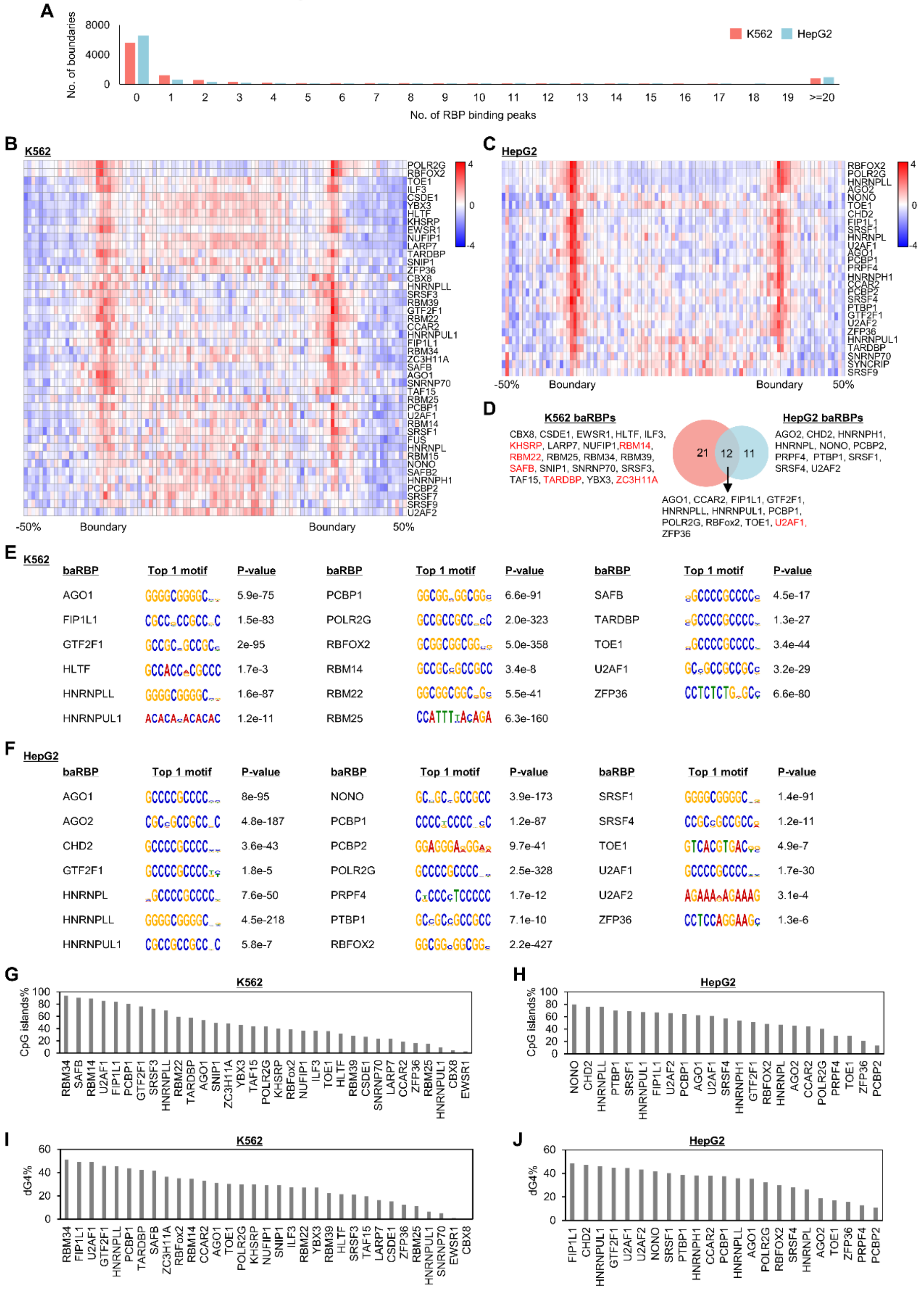

**Supplementary Figure S1. TAD boundaries are hotspots for RBP binding. (A)** The number of boundaries with the indicated number of RBP binding peaks. **(B-C)** Heatmaps showing the RBP ChIP-seq signals around TADs in K562 and HepG2 cells. The ±50% region flanking the TADs was used for profiling. **(D)** Overlapping of baRBPs in K562 and HepG2 cells. Known chromatin associated RBP (chrRBPs) are highlighted in red. **(E-F)** Enriched motifs of baRBP ChIP-seq peaks within TAD boundaries in K562 and HepG2 cells. **(G-H)** Bar plot showing the percentage of baRBP ChIP-seq peaks overlapped with CpG islands in K562 and HepG2 cells. **(I-J)** Bar plot showing the percentage of baRBP ChIP-seq peaks overlapped with dG4 ChIP-seq peaks in K562 and HepG2 cells.

**Sun Q, Zhou Q et. al. Suppl. Fig. S2**

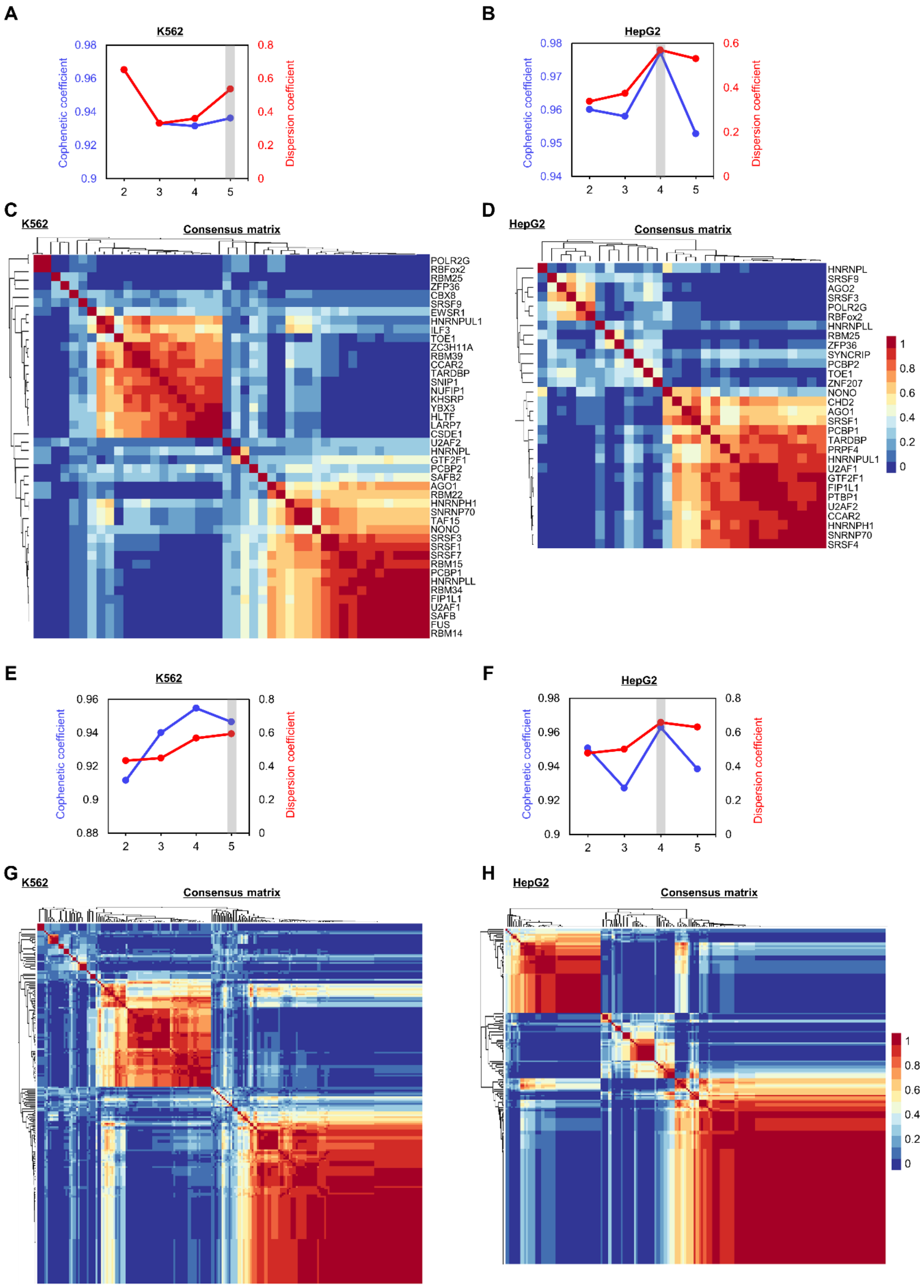

**Supplementary Figure S2. Network interaction of baRBPs and TFs at TAD boundaries. (A-B)** Criteria for estimating a maximal stable factorization rank in NMF analysis for baRBP clusters in K562 and HepG2 cells. Grey line: the factorization rank of 5 and 4 was chosen for the representation of RBP groups in Figure 2B-C. **(C-D)** The average connectivity matrix in NMF analysis in Figure 2B-C, showing multiple groups that are distinct from each other. **(E-F)** Criteria for estimating a maximal stable factorization rank in NMF analysis for baRBP-TF clusters. Grey line: the factorization rank of 5 and 4 was chosen for the representation of baRBP groups in Figure 2F-G. **(G-H)** The average connectivity matrix in NMF analysis in Figure 2F-G, showing multiple groups that are distinct from each other.

**Sun Q, Zhou Q et. al. Suppl. Fig. S3**

**A**

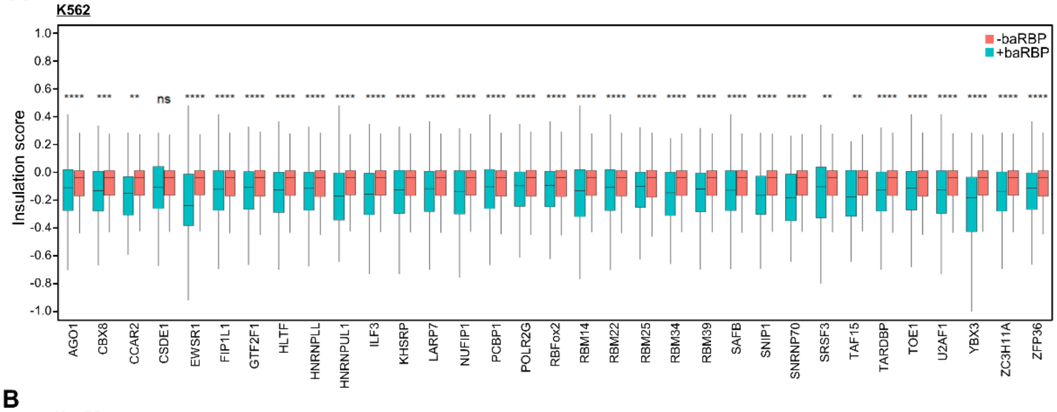


**B**

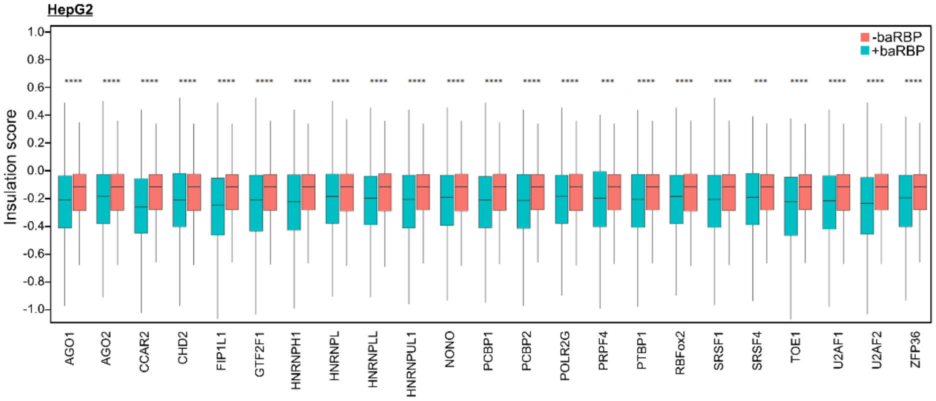

**Supplementary Figure S3. Comparison of insulation scores of +baRBP boundaries and -baRBP boundaries. (A-B)** Comparison of insulation scores of boundaries with and without each of the baRBP (33 in K562 and 23 in HepG2).

Sun Q, Zhou Q et. al. Suppl. Fig. S4

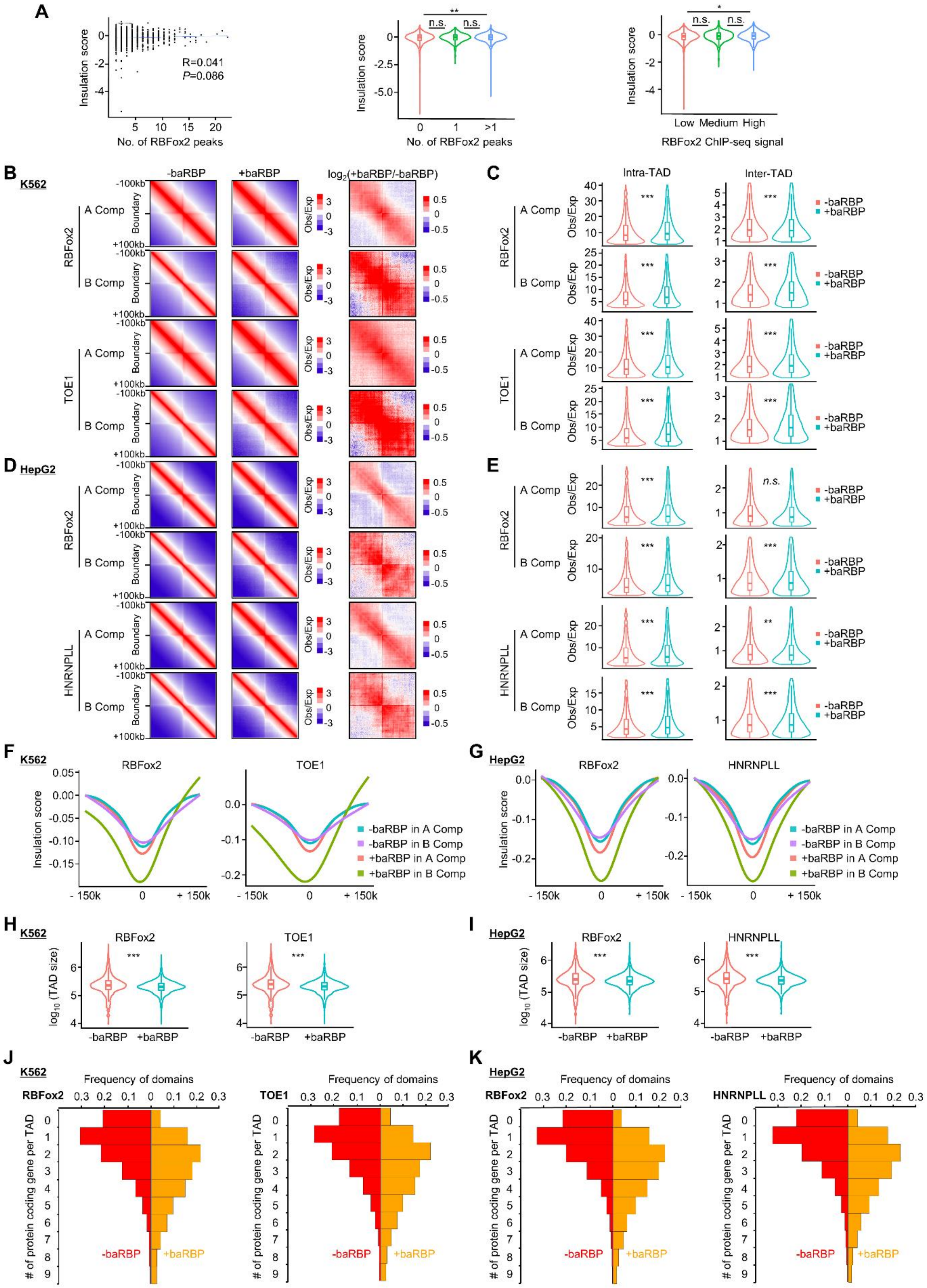

**Supplementary Figure S4. baRBP enrichment correlates with increased insulation strength of TAD boundaries.** (**A)** Correlation between insulation score and number of RBFox2 peaks (left and middle panels), and RBFox2 binding strength (low, medium or high) (right panel). **(B)** Aggregate analysis of interaction frequency around +baRBP (RBFox2 and TOE1) and -baRBP boundaries residing at A or B compartment in K562 cells. **(C)** Quantification of intra- or inter-TAD interaction frequency of data from **(B)**. **(D-E)** The above analyses were performed on baRBPs (RBFox2 and HNRNPLL) in HepG2 cells. **(F-G)** Comparison of insulation scores of the above +baRBP and -baRBP boundaries at A or B compartment in K562 and HepG2 cells. **(H-I)** Comparison of TAD size with or without the above +baRBP boundaries in K562 and HepG2 cells. **(J-K)** Comparison of numbers of genes per TAD with or without the above +baRBP boundaries in K562 and HepG2 cells.

**Sun Q, Zhou Q et. al. Suppl. Fig. S5**

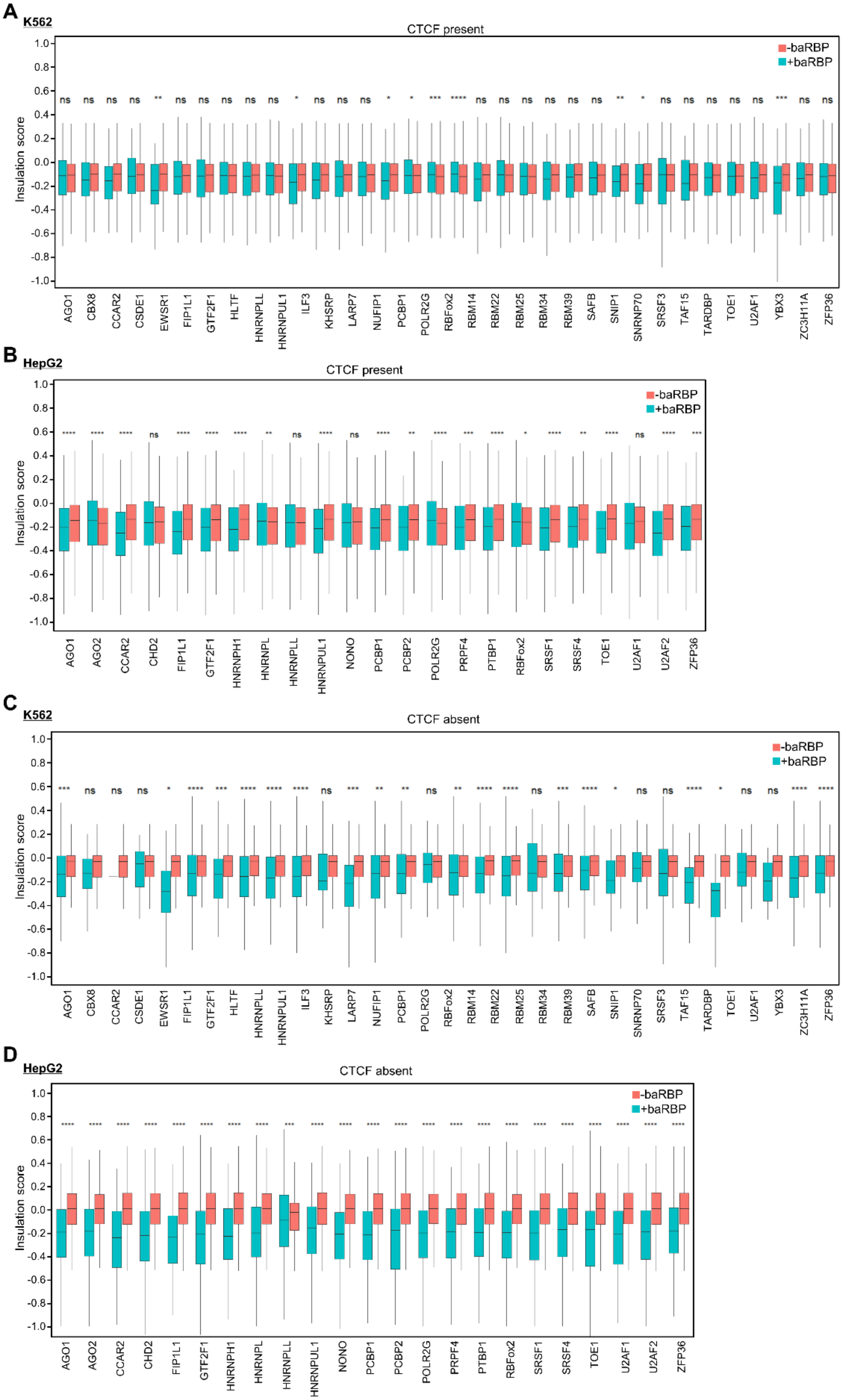

**Supplementary Figure S5. Comparison of insulation scores of +baRBP boundaries and -baRBP boundaries at CTCF present or absent boundaries. (A-B)** Comparison of insulation scores of boundaries with and without each of the baRBP (33 in K562 and 23 in HepG2) binding at CTCF present boundaries. **(C-D)** Comparison of insulation scores of boundaries with and without the above baRBP binding at CTCF absent boundary in K562 and HepG2.

**Sun Q, Zhou Q et. al. Suppl. Fig. S6**

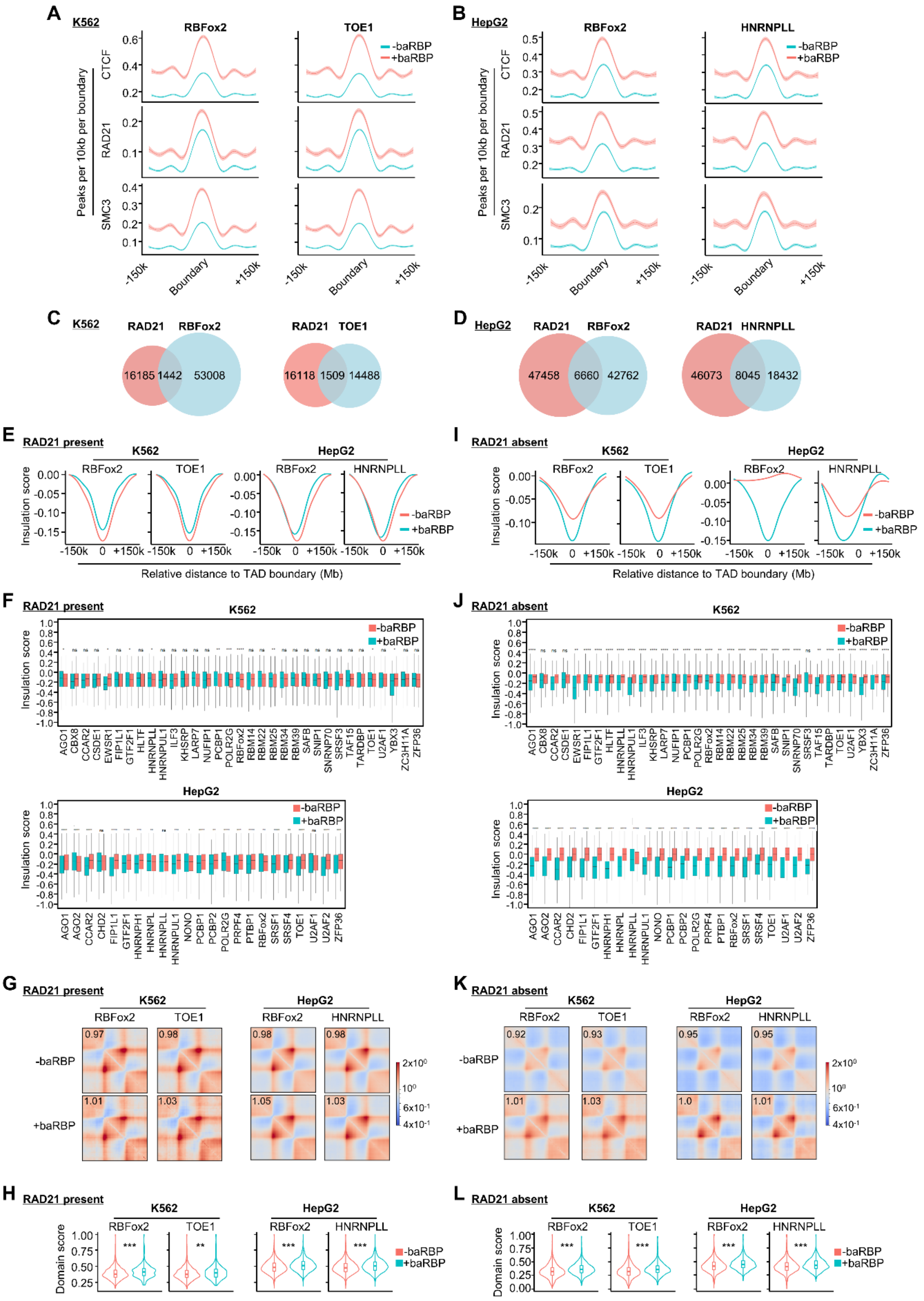

**Supplementary Figure S6. baRBPs may facilitate TAD organization independent of CTCF and cohesin binding. (A-B)** CTCF, RAD21, and SMC3 peak counts around +baRBP (RBFox2 and TOE1 in K562, RBFox2 and HNRNPLL in HepG2) or -baRBP boundaries. **(C-D)** Overlapping of RAD21 and representative baRBP (RBFox2 and TOE1 in K562; RBFox2 and HNRNPLL in HepG2) ChIP-seq peaks at TAD boundaries. **(E)** Comparison of insulation scores of +baRBP (RBFox2 and TOE1 in K562 and RBFox2 and HNRNPLL in HepG2) and -baRBP boundaries at RAD21 present boundaries. **(F)** Comparison of the insulation scores of boundaries with and without each of baRBP (33 in K562 and 23 in HepG2). **(G)** Aggregate domain analysis of TADs with +baRBP (RBFox2 and TOE1 in K562 and RBFox2 and HNRNPLL in HepG2) and -baRBP boundaries at RAD21 present boundaries. **(H)** Comparison of domain scores of the above TADs with +baRBP and -baRBP boundaries at RAD21 present boundaries. **(I-L)** The above analyses were performed at RAD21 absent boundaries.

**Sun Q, Zhou Q et. al. Suppl. Fig. S7**

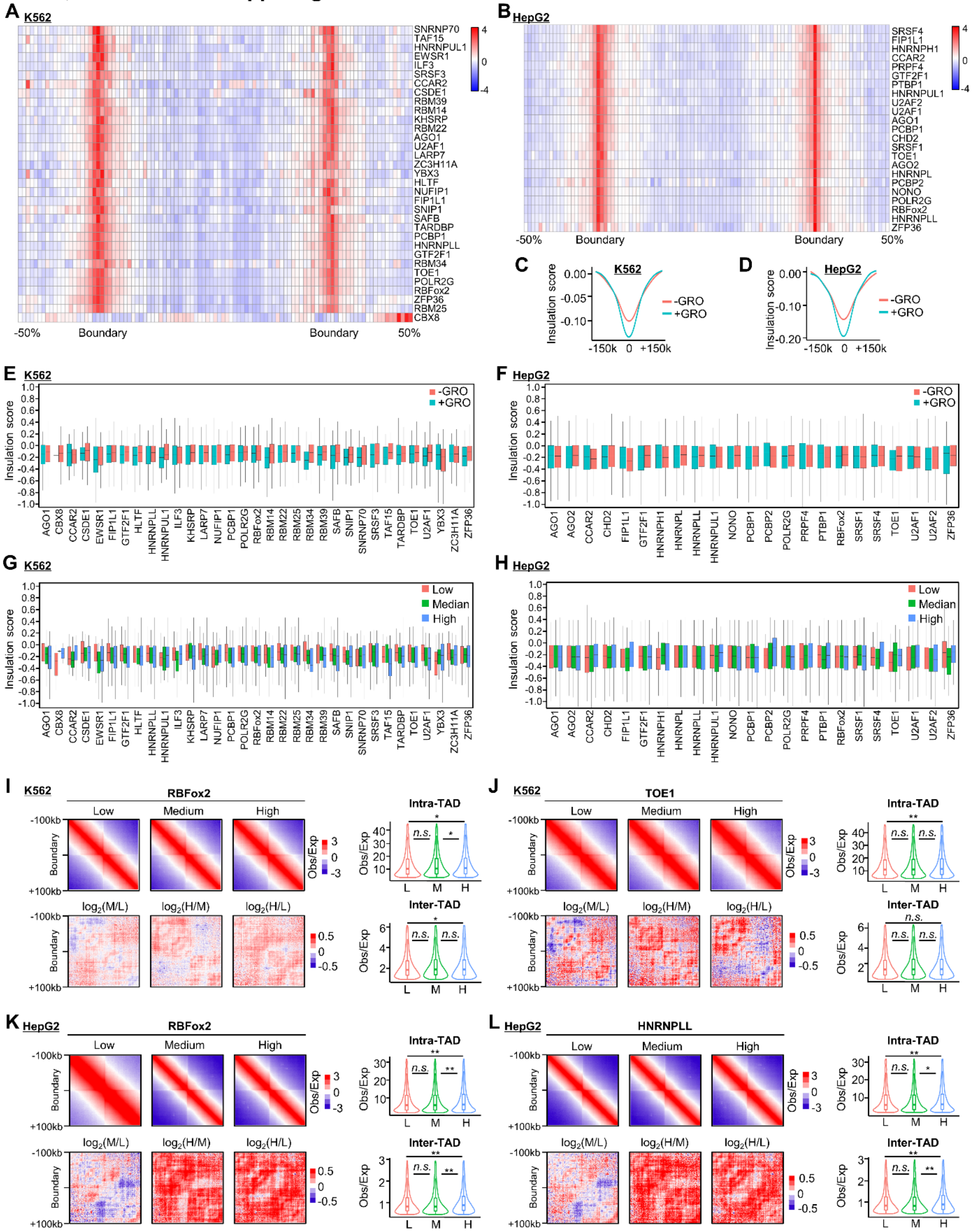

**Supplementary Figure S7. baRBP enrichment on TAD boundaries is correlated with active transcription. (A-B)** Heatmaps showing the GRO-seq signals around TADs with +baRBP (33 in K562 and 23 in HepG2) boundaries. The ±50% region flanking each TAD was used for profiling. **(C-D)** Comparison of insulation scores of TAD boundaries with or without GRO-seq signals. **(E-F)** Comparison of insulation scores of each of +baRBP (33 in K562 and 23 in HepG2) boundaries with or without GRO-seq signals. **(G-H)** Comparison of insulation scores of the above +baRBP boundaries with low, medium, or high level of GRO-seq signals. **(I-J)** Aggregate analysis of interaction frequency around +baRBP boundaries (RBFox2 and TOE1) with low, medium or high level of GRO-seq signals in K562. **(K-L)** The above analyses were performed on RBFox2 and HNRNPLL in HepG2.

**Sun Q, Zhou Q et. al. Suppl. Fig. S8**

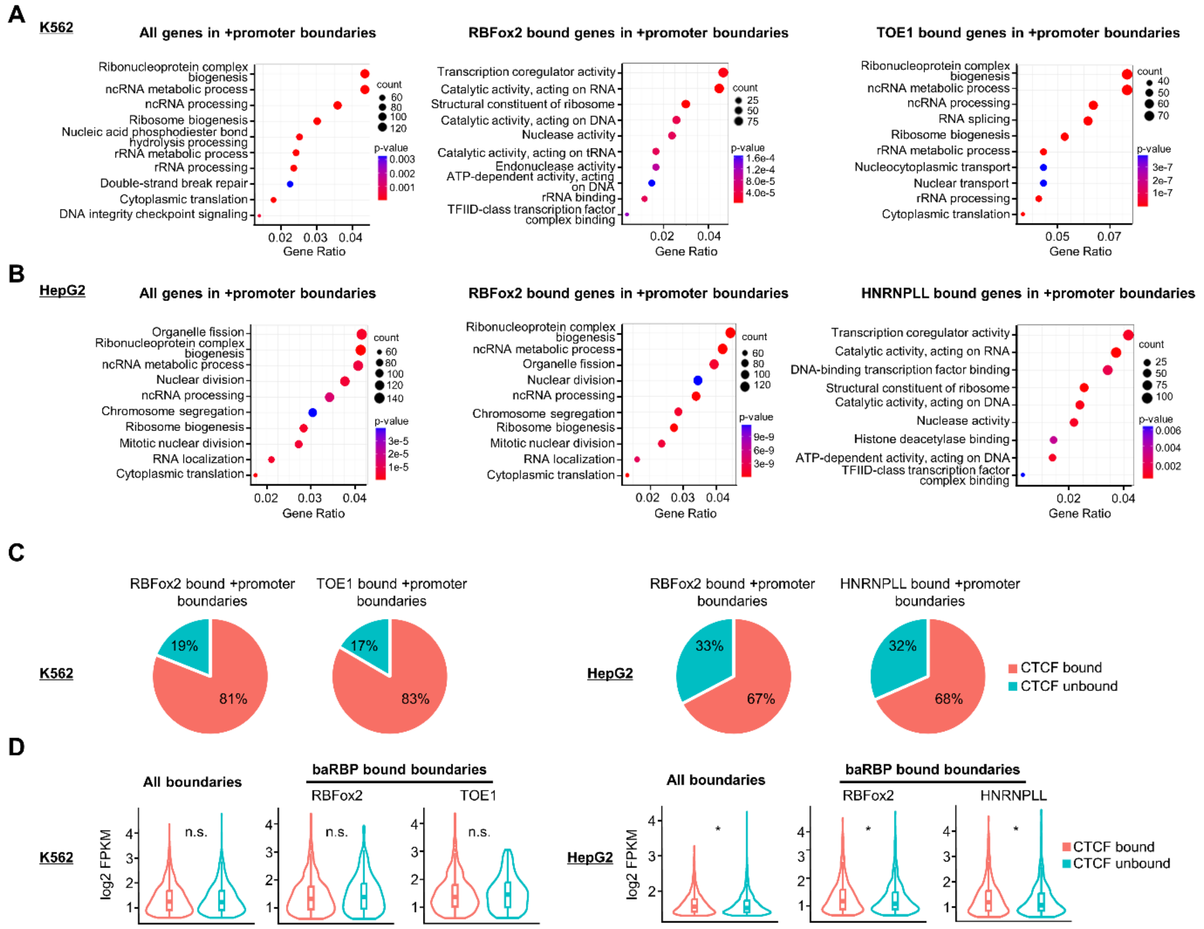

**Supplementary Figure S8. GO terms and expression of genes at +promoter boundaries. (A, B)** GO analysis of genes at +promoter TAD boundaries in K562 **(A)** and HepG2 cells **(B). (C)** Pie chart showing the percentage of baRBP bound +promoter boundaries that were occupied with CTCF in K562 (left panel) and HepG2 cells (right panel). **(D)** Comparison of expression of genes at baRBP bound +promoter boundaries with CTCF co-binding and those without CTCF binding in K562 (left panel) and HepG2 cells (right panel).

Sun Q, Zhou Q et. al. Suppl. Fig. S9

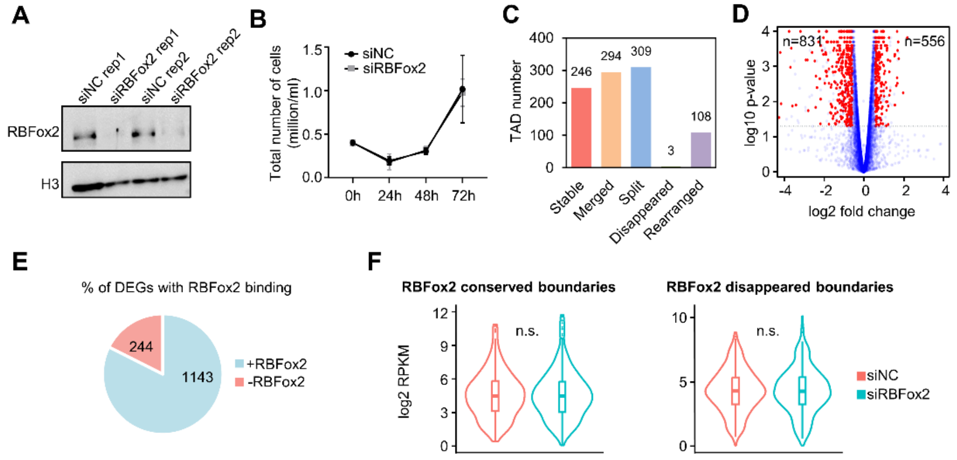

**Supplementary Figure S9. RBFox2 binding promotes TAD organization in K562. (A)** Western blotting assay to examine the RBFox2 expression in siNC and siRBFox2 sample. **(B)** Grow curve of K562 after RBFox2 depletion. **(C)** Bar plot showing the number of TADs with +RBFox2 boundaries that were stable, merged, split, disappeared or rearranged upon *RBFox2* depletion. **(D)** Volcano plot showing the differentially expressed genes (DEGs) after RBFox2 knockdown in K562 cells. **(E)** Pie chart showing the percentage of DEGs that overlapped with RBFox2 ChIP-seq peaks. **(F)** Comparison of expression of genes that located at RBFox2 conserved or disappeared boundaries upon RBFox2 knockdown.

**Sun Q, Zhou Q et. al. Suppl. Fig. S10**

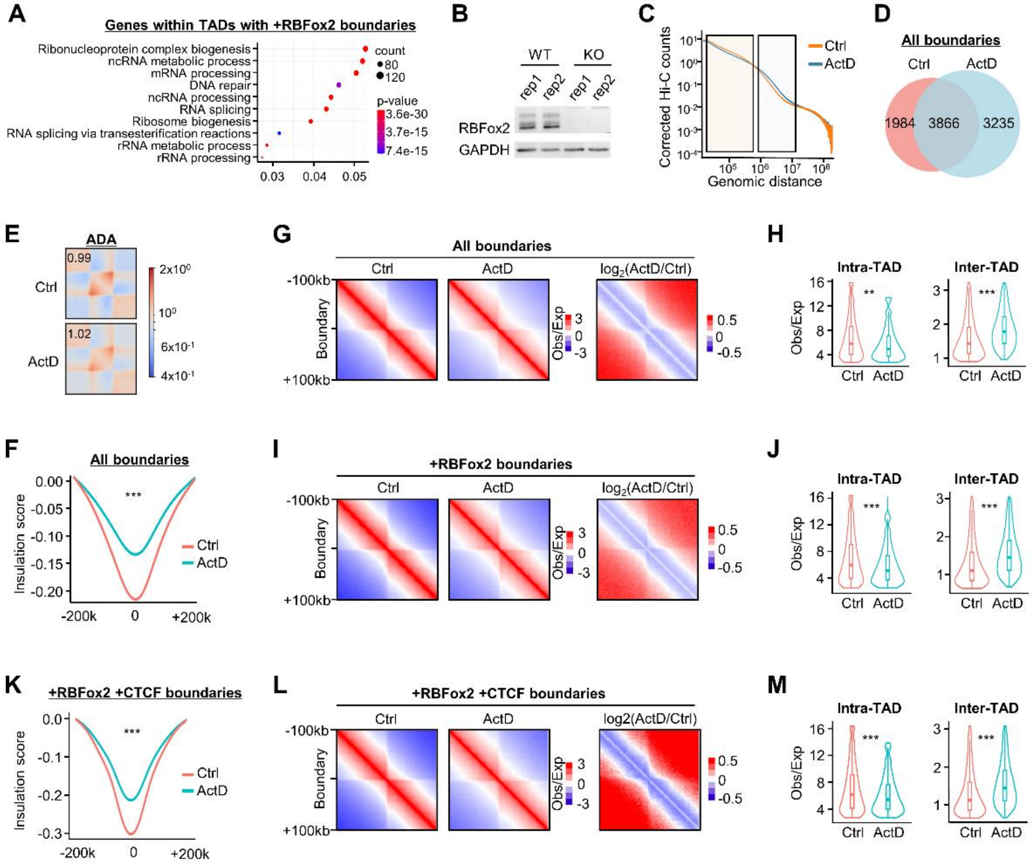

**Supplementary Figure S10. RBFox2 regulation of 3D genome is relevant in mouse myoblast differentiation. (A)** GO analysis of genes residing in TADs with +RBFox2 boundaries in MBs. **(B)** Western blotting showing the protein expression of RBFox2 in the wild type (WT) and RBFox2 knockout (KO) C2C12 cells. **(C)** Contact frequency as a function of genomic distance along the whole genome in Ctrl vs ActD treated MBs. **(D)** Dynamic change of all boundaries upon ActD treatment. **(E)** ADA analysis in Ctrl and ActD treated MBs. **(F)** Comparison of insulation scores of all boundaries in Ctrl and ActD treated MBs. **(G)** Aggregate analysis of interactions around all boundaries in Ctrl and ActD treated MBs. **(H)** Comparison of inter- and intra-TAD interactions around all boundaries in **(G)**. **(I-J)** The above analyses were performed on +RBFox2 boundaries in Ctrl and ActD treated MBs. **(K)** Comparison of insulation scores of +RBFox2+CTCF boundaries in Ctrl and ActD treated MBs. **(L)** Aggregate analysis of interactions around +RBFox2+CTCF boundaries in Ctrl and ActD treated MBs**. (M)** Comparison of inter- and intra-TAD interactions around +RBFox2+CTCF boundaries in **(L).**

## 2. Supplementary Tables

Supplementary Table S1. Sources of high throughput sequencing data used in the study.

Supplementary Table S2. Lists of TADs and TAD boundaries identified in K562 and HepG2 cells.

Supplementary Table S3. Network analysis of baRBPs on TAD boundaries.

Supplementary Table S4. Analysis of TADs with baRBP binding boundaries.

Supplementary Table S5. Analysis of RBP co-binding with CTCF and cohesin.

Supplementary Table S6. Analysis of GRO-seq signals at baRBP enriched boundaries.

Supplementary Table S7. baRBP ChIP-seq peaks at promoter containing boundaries.

Supplementary Table S8. Analysis of TAD and TAD boundary in C2C12 MB and MT cells.

Supplementary Table S9. Analysis of the effect of ActD treatment on 3D genome.

Supplementary Table S10. Sequences of oligos used in this study.
